# A systematic review of lived experiences of people with polycystic ovary syndrome highlights the need for holistic care and co-creation of educational resources

**DOI:** 10.3389/fendo.2022.1064937

**Published:** 2022-12-02

**Authors:** Gar Mun Lau, Mirna Elghobashy, Mansi Thanki, Shirley Ibegbulam, Pallavi Latthe, Caroline D. T. Gillett, Michael W. O’Reilly, Wiebke Arlt, Antje Lindenmeyer, Punith Kempegowda, PCOS SEva Working Group

**Affiliations:** ^1^ College of Medical and Dental Sciences, University of Birmingham, Birmingham, United Kingdom; ^2^ School of Pharmacy, University of Birmingham, Birmingham, United Kingdom; ^3^ Birmingham Women’s Hospital, Birmingham Women’s and Children’s National Health Service (NHS) Foundation Trust, , Birmingham, United Kingdom; ^4^ Institute of Metabolism and Systems Research, University of Birmingham, Birmingham, United Kingdom; ^5^ Royal College of Surgeons in Ireland (RCSI) University of Medicine and Health Sciences, Dublin, Ireland; ^6^ Department of Endocrinology, Queen Elizabeth Hospital, University Hospitals Birmingham National Health Service (NHS) Foundation Trust, Birmingham, United Kingdom; ^7^ Institute of Clinical Sciences, University of Birmingham, Birmingham, United Kingdom

**Keywords:** polycystic ovary syndrome, PCOS, lived experience, expectations, perspectives, satisfaction, culturally appropriate resources

## Abstract

**Introduction:**

PCOS-related literature is mostly dominated by the medical perspective. However, the condition’s lifelong, far reaching, and multifaceted impacts highlight the importance to gain the perspectives from those with PCOS. Therefore, we performed a systematic review to explore the current literatures and gaps around the experiences and perceptions of those living with PCOS

**Method:**

A comprehensive search of seven electronic databases was conducted between July and October 2021. A total 34 from 1615 screened articles were included in this systematic review and subsequently coded using NVivo 12 software. The quality of individual studies was assessed by adaptation to the Critical Appraisal Skills Program (CASP) quality assessment tool.

**Results:**

Five domains were generated from the data: Signs/Symptoms, Diagnosis, Management, Perceptions, Resources and Improving Outcomes. Dissatisfaction surrounding the experience of diagnosis was common. Concerns surrounded perceived lack of knowledge from healthcare professionals and delays in diagnosis. Individual studies on adults and adolescents shared similar feelings. The consensus was found to be that current management was vague and generalised. Symptoms such as hirsutism, obesity, irregular menstruation challenge personal and societal expectations of femininity. Online PCOS resources are popular amongst those with PCOS but most of them lack evidence. A call for more culturally specific resources was found to be common ground amongst those with PCOS.

**Conclusion:**

Overall dissatisfaction amongst adults and adolescents regarding their diagnostic journey of PCOS. Tailored and culturally specific PCOS advice and management is necessary and can be achieved through co-creation of resources between healthcare professionals and those with PCOS.

**Systematic review registration:**

https://www.crd.york.ac.uk/prospero/, identifier CRD42021272371.

## 1. Introduction

Polycystic Ovary Syndrome (PCOS) is a common endocrine condition affecting up to 20% of women of reproductive age globally (1). Clinical features include hyperandrogenism, oligo/anovulation and the polycystic appearance of the ovaries on ultrasound. PCOS is associated with weight gain, acne, hirsutism, and impaired fertility due to chronic oligo-anovulation (2–4). In addition to androgen excess, insulin resistance is a central feature and women with PCOS have been shown to be at increased risk of type 2 diabetes, hypertension, and fatty liver disease. There are no curative options currently for PCOS, therefore treatment aims to alleviate symptoms and prevent long-term complications, including cardiovascular events, and endometrial cancer (5).

PCOS-related literature is mostly dominated by the medical perspective (6). However, given the condition’s lifelong, far reaching, and multifaceted impacts, the perspective of women living with PCOS must be highlighted to tailor support. They are expected to largely self-manage their condition through lifestyle changes and medications and therefore, adequate education, information and coping skills are important to achieve sustainable management in the long term. Previous studies have highlighted that many people with PCOS report a lack of adequate information, in addition to negative encounters with healthcare professionals, including a lengthy diagnostic process and a lack of clinical, educational, and emotional support (7–9). Understanding the experiences and concerns of those with PCOS is necessary to provide appropriate support and improve their quality of life. This review aims to identify and summarise the lived experiences and perspectives of those with PCOS from the available literature.

## 2. Methods

This qualitative thematic synthesis includes literature identified in databases in a population of people with PCOS and were considered to meet any of the following criteria:

1. Explore the experiences and perceptions of those living with PCOS
2. Assess the knowledge and educational needs of those with PCOS
3. Discuss strategies to deliver health knowledge in those with PCOS

### 2.1. Inclusion and exclusion criteria

Studies were included if they included: (i) participants of any age group with a suspected/confirmed diagnosis of PCOS, (ii) original research studies, including qualitative studies, of people with PCOS and (iii) informed consent of study participants and ethics committee approval.

In adults, PCOS is suspected if a woman has one or more clinical features of:

* Infrequent or no ovulation manifested as infertility, oligomenorrhoea, or amenorrhoea.
* Hyperandrogenism manifesting as hirsutism and acne.

In adolescents, suspect PCOS if the girl has signs and symptoms of hyperandrogenism (such as acne and hirsutism), and irregular menstrual cycles

PCOS is also suspected if there is a family history of PCOS, or indirect evidence of insulin resistance such as obesity, acanthosis nigricans (10)

PCOS is diagnosed in adults if two of three of the following criteria are present, provided other causes of menstrual disturbance and hyperandrogenism are excluded (4, 11):

* Infrequent or no ovulation (usually manifested as infrequent or no menstruation).
* Clinical and/or biochemical signs of hyperandrogenism (such as hirsutism, acne, or elevated levels of total or free testosterone).
* Polycystic ovaries on ultrasound scan, defined as the presence of 12 or more follicles (measuring 2–9 mm in diameter) in one or both ovaries and/or increased ovarian volume (greater than 10 cm^3^).

In adolescent girls, both hyperandrogenism and irregular menstrual cycles are required for a diagnosis of PCOS.

There were no restrictions on age, ethnicity, or health status information. The following study types were included for review: qualitative studies, randomised controlled trials, non-randomised controlled trials, observational studies - prospective and retrospective cohort studies, case-control studies, and cross-sectional studies. Studies that described PCOS without any clinical data, review articles, case reports, case series and papers that involved animal studies were excluded from this review.

### 2.2. Search strategies and selection

Preliminary searches were conducted by reviewer GL and ME, from August 2021 to October 2021. Online electronic databases (PubMed, Medline and Embase) were searched for literature published up to August 2021; the other databases (APA PsychInfo, Web of Science and CENTRAL) included literature published up to October 2021. Searches had no language, publication, or geographical restrictions applied. Search terms used in this study are as follows: PCOS, Polycystic ovary syndrome, polycystic ovarian disease, Stein-Leventhal syndrome, Education, Health Education, Information, Health Information Exchange, Consumer Health Information, Access to Information/or Information Seeking Behaviour/or Information Literacy, Access to Information/or Information Seeking Behaviour/or Information Literacy, Resources, Health resources, Video, Video-Audio Media, Lived Experiences, opinion, perspective, viewpoint, comment attitude, knowledge, understanding, comprehension, Patient Medication Knowledge/or Knowledge/or Health Knowledge, Attitudes, Practice/or knowledge. A detailed breakdown of searches in various databases is included in **Supplementary Tables 1–6**.

**Figure 1:**
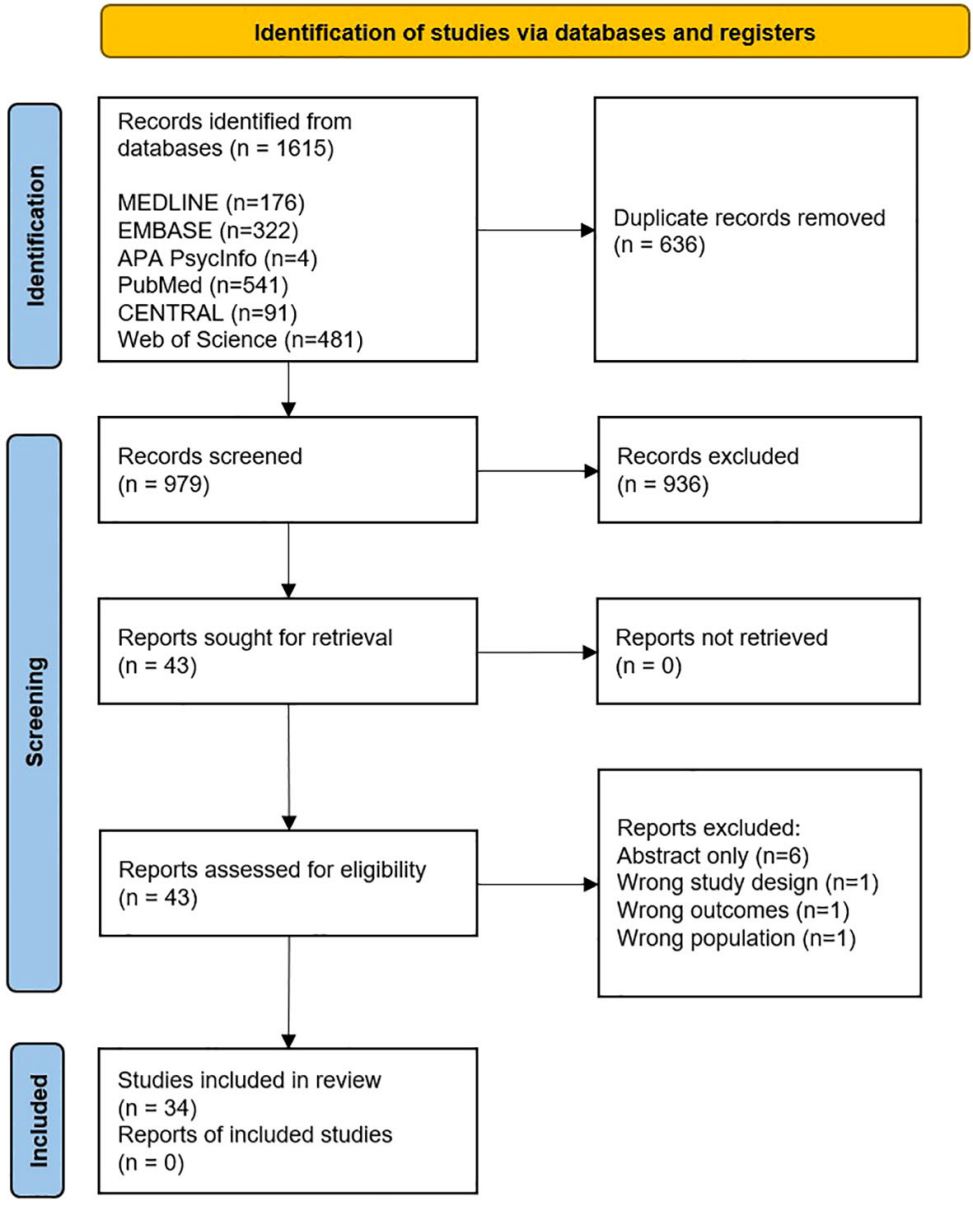
PRISMA chart for the study.

Initially a total of 1615 studies were identified, reduced to 979 after duplicates were removed. Author GL and ME screened titles and abstracts of search results according to the inclusion and exclusion criteria, this excluded a further 936 papers. Full paper screening was conducted and issues regarding paper relevance were resolved through discussion with senior supervisor PK. The Critical Appraisal Skills Programmer (CASP) qualitative studies and systematic review checklist were used to appraise the relevance and validity of the full texts identified. A final total of 34 studies were included in this narrative review (**Table 1**). The PRISMA chart (Preferred Reporting Items for Systematic Reviews and Meta-Analyses) for the study is described in **Figure 1**.

**Table 1:** Studies included in this systematic review.

No.	Study	Year	Country	Study Type	Participants	Recruited from
1	Sills et al	2001	USA	Descriptive measurements	657 unique respondents	Free public access site OBGYN.net
2	Snyder	2006	USA	Phenomenological Analysis	12 premenopausal women	Women's health practice in northeast US
3	Avery & Mayer.	2007	Australia	Framework content analysis	10 women	Previous PCOS national study and University of Adelaide's Department of Obstetrics and Gynaecology
4	Humphreys et al.	2008	UK	Descriptive analysis	53 women	Endocrinology department, Middlesex Hospital, London
5	Percy et al.	2009	UK	Qualitative interviews	13 women	Support group at public UK hospital
6	Colwell et al	2010	Canada	Descriptive statistics	43 women	Poster advertisement and previous patients involved in a clinical study
7	Jones et al	2011	UK	Qualitative thematic Analysis	15 women	Outpatient gynaecology clinic in Sheffield and Leeds
8	Weiss & Bulmer	2011	USA	Qualitative phenomenological study	12 women (aged 18-23), (students)	University campus in Connecticut, a private, public and community college
9	Holbrey et al.	2013	UK	Essentialist Thematic Analysis	50 women	UK based support group
10	Tomlinson et al.	2013	UK	Qualitative analysis	18 women	Poster advertisements or referrals by GPs or hospital consultants
11	Gibson-Helm et al	2014	Australia	Descriptive statistics	210 women	Poster advertisements from general or health media, 18% from a national women’s health website and 13% from a support group website
12	Williams et al	2015	UK	Thematic Analysis	10 participants	Support group on Facebook
13	Williams, et al.	2016	UK	Thematic Analysis	9 women	UK Verity Charity
14	Gibson-Helm, et al.	2017	Multinational	Cross-sectional study	1385 women	PCOS Support Groups PCOS Challenge and Verity
15	Tomlinson et al.	2017	UK	Qualitative thematic Analysis	11 focus groups with 2-6 women in each	Poster advertisement and GP and secondary care clinic referrals.
16	Hadjiconstantinou et al.	2017	UK	Qualitative analysis	12 ethnically diverse women	Local Leistershire hospital by advertisement or initial approach at outpatient clinics, and three local women who approached the research team after learning about the study
17	Holton, et al.	2018	Australia	Cross-sectional study	1543 women (113 with PCOS)	Australian Electoral Commission
18	Sharma.& Mishra.	2018	India	Interpretative phenomenological analysis	35 women	Outpatient department of government hospital in Jammu, India.
19	Lin et al	2018	USA	Cross-sectional study	332 women	Posters on Twitter, Facebook, Reddit and Research Match for women with PCOS and without on ClinicalTrials.gov
20	Gibson-Helm et al	2018	Australia	Systematic search and narrative review	35 studies	Scopus, ProQuest, Central, all EBM Revies, CINAHL Plus, PsycINFO and Ovid Medline
21	Copp et al.	2019	Australia	Framework content analysis	26 women	Facebook
22	Young et al	2019	USA	Qualitative descriptive	7 adolescents with PCOS and 8 parents	Adolescent medicine clinic in large city in south central US *via* clinicians or posters.
23	Carron, et al.	2020	USA	Leininger's qualitative analysis	13 key informants	Tribal reservation in Western America
24	Hoyos, et al.	2020	USA	Analysis of search trends	759 respondents	Google and Storybase
25	Authier et al.	2020	France	Inductive qualitative analysis	785 comments by 211 women	French speaking internet forum
26	Wright et al.	2020	USA	Low inference content analysis	95 stories	Online support webpage Soul Cysters
27	Ee et al.	2020	Australia	Thematic Analysis	10 women	Advertisements on Facebook and relevant women's health organisation pages.
28	Hillman et al	2020	UK	Mixed methods	323 women completed surveys; 11 women conducted interviews	GP referrals and surveys through Mumsnet and Facebook charity pages of Verity, Ladies Circle and Fertility Friends Network
29	Pirotta et al	2021	Australia	Phenomenology qualitative research design	20 women	Facebook, Instagram, Polycystic Ovary Association of Australia (POSAA)
30	Kurdi et al	2021	Jordan	Quasi-experimental	68 participants	Rufaida College and Nusabia College
31	Lim et al.	2021	Australia	Qualitative study	10 women	Facebook
32	Copp, et al.	2021	Australia	Qualitative study	36 clinicians and 26 women	Advertising *via* rel. Royal Australian College of General Practitioners and Endocrine Society of Australia, active and passive snowballing, and contacting a random sample of endocrine and gynaecology teams across Australia.
33	Douglas et al	2021	USA	Descriptive design	12 women, university students	Public university in southern US
34	Jena et al	2021	India	Descriptive statistics	965 young women	Obstetrics and Gynaecology department, AIIMS, Bhubaneswar

### 2.3. Data synthesis and analysis

Data were analysed using NVivo 12 Software (Mac and Windows) using Thomas and Harden’s thematic synthesis approach (12). Inductive analysis is a type of thematic analysis where the data is coded without trying to fit it into a pre-existing coding frame or the researcher’s analytic preconceptions. This type of analysis provides a rich and detailed, yet complex account of data (13). Thematic synthesis was chosen as it allows for common themes to be drawn from diverse studies with ease, to directly educate and inform patients and healthcare professionals (14). Most studies were qualitative or mixed methods studies (15), but there were some case studies (6) and a few other study designs. Inductive analysis stages included (i) extraction of information and coding of findings, (ii) grouping of ‘codes’ according to themes being discussed (iii) analysis of codes and subsequent themes to identify and capture findings from the review (16).

Stage (i) and (ii) of the analysis was performed by authors SI and MT, who were blind to the title of the review, synchronously and independently. Papers were downloaded into PDF formats and read in their entirety before coding commenced and codes were created from abstract to conclusion. All codes grouped under themes were re-read to ensure relevance to theme. Stage (iii) was analysed by author GL and overseen by senior supervisors PK and AL with any disagreements on the findings resolved by discussions between them. From this, domains with subsequent themes were formed.

The authors who did the coding were undergraduate students who did not have any experience in scientific research at that time. Therefore, we chose thematic analysis as it is easily grasped and can be relatively quick to learn, as there are few prescriptions and procedures. While thematic analysis is flexible, this flexibility can lead to inconsistency and a lack of coherence when developing themes derived from the research data (17). Therefore, we decided to blind our colleagues from the objective of the study and requested them to independently code the included articles. Their codes were then merged to synthesise the themes, thus enhancing the quality and reliability of the emerging themes.

### 2.4. Rigour

The literature search, coding of themes, and development of domains were done independently by different members of the research team, therefore, reducing bias and increasing reproducibility of data. Researchers MT and SI were blind to the research question before coding reducing investigator bias. MT and SI both coded all full-text papers in this study with their codes later merged, reducing confirmation bias. The entire process was overseen by senior researchers PK and AL experienced in qualitative research.

## 3. Results

**Figure 2:**
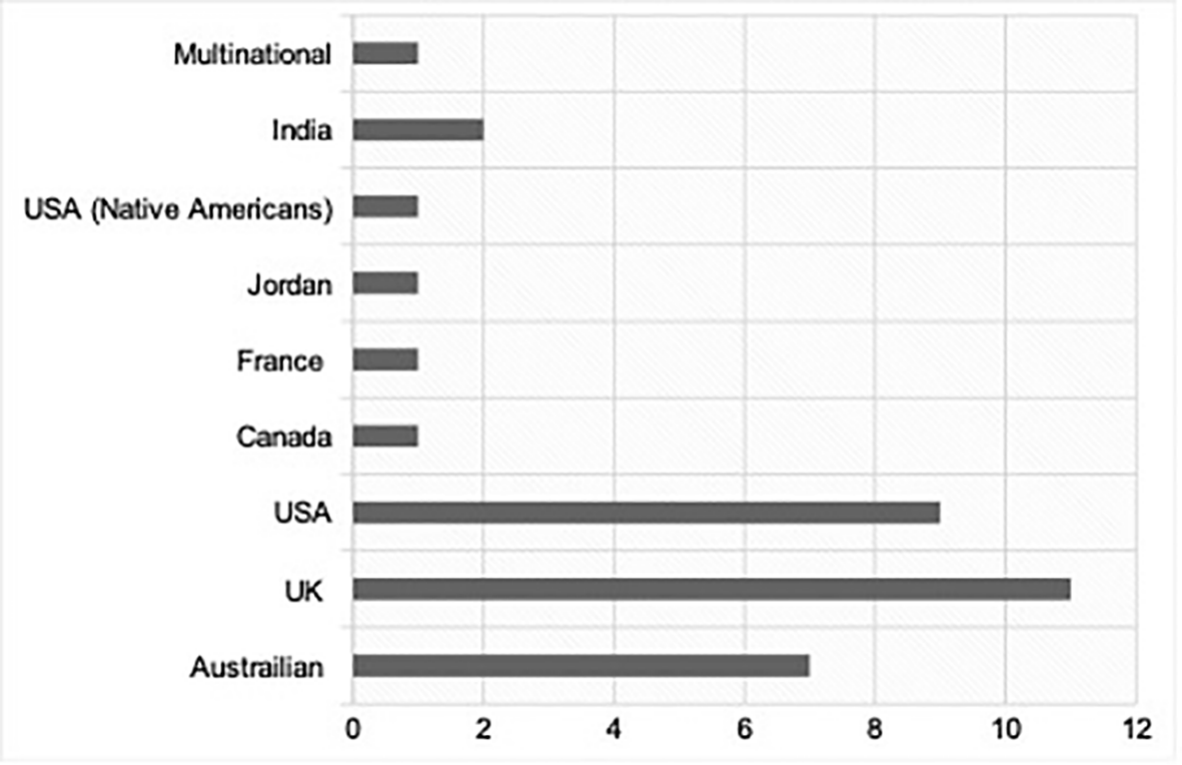
Chart displaying number of studies per country/ respective community.

The articles identified in this review were published between 2001 and 2021, detailing international populations of people with PCOS; see **Figure 2** for a summary of studies. Most studies collected data in high-income countries which predominantly speak English including the UK, USA and Australia. Most study types were qualitative in nature and generally involved either individual semi-structured interviews, focus groups or open-text questionnaires. Some data collection occurred online, which was particularly useful in enabling researchers to reach international and more socially isolated groups (18). In one study, participants were recruited *via* social media (19). Other recruitment methods include newspapers and flyers (20), hospital clinics and GP surgeries (6, 15, 21), support groups (22) and university campuses (23). Participants in studies were usually described as ‘women’; however, we understand that not all PCOS sufferers identify as such (and therefore we have decided to use ‘people with PCOS’ in this article). It is unclear whether participants in the literature self-identified as women or whether other data such as patient records were used. Thematic analysis identified the following five domains: Signs and Symptoms of PCOS, Diagnosis of PCOS, Management of PCOS, Perceptions, and Resources and Improving Outcomes. We describe each of these below:

### 3.1. Signs and symptoms

This domain focused on the common symptoms that the participants experiences, including which symptoms prompted them to seek care. This section also includes details of clinical signs observed by clinicians during their diagnosis and management.

#### 3.1.1. Menstrual issues

Irregular menstruation was the symptom for which most people with PCOS sought medical attention (24, 25). Some reportedly suffered anxiety due to uncertainty as to when their next period will be (26). In young adults the inconvenience of irregular menstrual bleeding also had major consequences on their social life. Ee et al., found that those aged 18 to 25 were more likely to identify irregular menstruation as a problem compared to those aged 26-35 (27).

#### 3.1.2. Subfertility

Many people with PCOS reported fear of being unable to conceive children. These fears were often conveyed as emotions of sadness, anger and humiliation (28). Fears of childlessness affected not only adults, but also adolescents with PCOS, who were three times more likely to express concerns with future fertility than those without PCOS of the same age (29). However, the study by Jones et al. found that many adolescents were not overly concerned about their chances of fertility as it was not deemed an imminent priority (26). In some cultures, subfertility could result in greater impacts on the community, personal economics, the law and family dynamics, and religious and spiritual responsibilities than in traditionally Western societies (30). A study conducted in Iran found infertile women were more likely to be subjected to domestic violence; they were also isolated from social events and family (31).

#### 3.1.3. Mental health

Deeks et al. (3) reported that symptoms of depression and anxiety affected women with PCOS particularly those reporting subfertility. In a study by Coffey et al., those with PCOS experienced poorer health related quality of life compared to those associated with other chronic conditions such as asthma, epilepsy and coronary heart disease (32). Ee et al. found that acne and facial hair were major contributors to poor mental health in some women (27). Himelein and Thatcher reported body dysmorphia was very high in nulliparous women with PCOS (33). Jones et al. noted the concerns regarding feminine identity and attractiveness were particularly high in adolescents with PCOS affected by obesity and hirsutism (26).

#### 3.1.4. Weight

A systematic review by Lim et al. (34) found that people with PCOS are more likely to suffer from excess weight gain and obesity than those without the condition. Young et al. (35) reported weight gain and difficulty losing weight were the most troubling health related concerns. Many struggled with the disparity between their actions and desired outcomes, which caused them significant distress. Douglas et al. (36) found that the majority of study participants were dissatisfied with their weight and wanted to lose weight. Synder (37) reported that some women who were overweight expressed guilt despite being aware that it is an associated symptom of PCOS.

#### 3.1.5. Hirsutism

In a multi-ethnic qualitative study by Hadjicontantinou et al. (38), many people with PCOS describe hirsutism as having a negative impact on their social identity as a woman. Some study participants likened this to “turning into a boy” and looking “like a man” (18, 25).

### 3.2. Diagnosis of PCOS

This domain related to the experiences of those with PCOS during their initial diagnosis and their perceptions of their diagnostic journey.

#### 3.2.1. Barriers to diagnosis

A study by Avery and Braunack-Mayer (7) found that most people with PCOS believe their healthcare practitioner did not know enough about PCOS as a condition. Copp et al. identified the heterogeneity and wide variation in presentation of PCOS as a barrier, with differentiation of PCOS-related features from normal variability in the general population, or other conditions such as hypothyroidism, were reported to be difficult for healthcare professionals (39). Tomlinson et al. (6) reported delay in specialist referral and lack of evidence-based tools as a barrier to a timely diagnosis. Uncertainties and inconsistencies with the current diagnostic criteria ultimately create challenges in the diagnostic process (40, 41).

#### 3.2.2. Experience of diagnosis

Frustration regarding the process of diagnosis was common in a study of 657 women with PCOS by Sils et al. (42). Dissatisfaction stemmed from perceptions of healthcare professionals lacking adequate knowledge of PCOS, therefore negatively impacting on their ability to provide appropriate information, facilitate positive health-related behaviours, and manage health outcomes. A similar finding was found in a systematic review by Gibson-Helm et al. (43).

Delays in diagnosis impacted experiences of diagnosis greatly. The study by Copp et al. (25) found time since diagnosis varied from 6 months to 27 years (median 6.5 years) and time from first seeking medical attention to receiving diagnosis ranged from 1 week to 8 years. In the study by Gibson-Helm et al. (8), the diagnosis took >2 years in24% of women and 39% saw three or more health professionals before a diagnosis was made. The latter group was negatively associated with diagnosis satisfaction. Hillman et al. (44) reported the delay in diagnosis was associated with a lack of trust by women with PCOS towards their primary care providers. In the study by Jones et al. (26), adolescents were dissatisfied with the slow referral process to secondary specialists. Once referred, they found hospital consultations brief and infrequent, and therefore felt unable to share their concerns with qualified healthcare professionals.

Some people with PCOS found their diagnosis beneficial. Copp et al. (25) found that receiving a diagnosis provided women an explanation for symptoms, validating their experiences which had been dismissed in the past by healthcare professionals or others. Other beneficial outcomes of receiving a diagnosis include: relief of stress; increased understanding of the condition, solidarity with others with PCOS; empowerment to take control of their own care; and motivation to implement change in their lifestyles (18, 43). Synder (37) reported a diagnostic label provided relief to those with PCOS that their symptoms could be attributed to factors outside of their control. Furthermore, early diagnosis allows those with PCOS to engage in lifestyle modification and better manage associated metabolic complications (24, 45).

### 3.3. Management of PCOS

Lifestyle changes including exercise and dietary changes were described as most common and often considered first-line. Some studies reported that a proportion of patients with PCOS did not receive any lifestyle advice (8, 44). A study of 68 women with PCOS by Colwell et al. (46) found that receiving some, albeit inadequate lifestyle advice regarding weight loss and wanting more appropriate help was a common experience. A study of 53 women with PCOS by Humphreys and Costarelli (47) reported that even among those who did receive advice, it was considered too general and vague.

Management of excess hair growth was rarely stated in the literature, with patients usually self-managing through hair removal creams, waxing, and shaving. Few studies acknowledged the potential for private laser hair removal procedures (18, 38, 48).

Oral contraceptives were the most use pharmacological management for PCOS. They were usually prescribed for the management of menstruation, acne and excess hair growth (4). Use of oral contraceptives showed a great variation in experiences, with some positive, and others negative (38, 49). The negative experiences mostly referred to side effects associated with oral contraceptive use such as secondary amenorrhoea (6).

Many people with PCOS complained of long-standing medication use and over-prescription without any other forms of support. Ee et al. and Hillman et al. (27, 44) have reported that some women in their cohorts took medication for longer than a decade. Several studies highlighted that women with PCOS felt poorly informed about the medications prescribed to them and were not receiving communications about potential alternative therapies (6, 23, 48). A study of 657 women with PCOS by Sills et al. (42) found that 99% patients with PCOS were interested in complementary therapies. Reasons stated for preference of complementary therapies were side effects associated with traditional interventions, dislike of pharmacological agents and desperation for treatment due to ineffectiveness of conventional medication (22, 27, 44). Copp et al. (49) reported that some healthcare professionals understood the increasing demand and curiosity of complementary treatments and supported them provided they were safe and effective although many had fears due to their unproven and unverified nature being advertised to the public.

#### 3.3.1. Barriers to management

Although some literature has suggested that adherence to lifestyle modification advice is difficult for those with PCOS (50), others have found that in fact women with PCOS are more likely to attempt most weight loss approaches, such as “various dietary patterns, exercise, medication, meal replacements, commercial weight loss programs and nonconventional methods” (51, 52).

In a study of 20 women through semi-structured interviews, Pirotta et al. (19) reported distractions in daily life was a barrier in following lifestyle advice. This includes a lack of time due to familial, personal and career commitments, easy access to processed foods and prioritising leisure events which disrupt their lifestyle patterns. Many participants in this study reported loss of motivation after noticing no improvements in weight initially in their weight loss endeavours.

Costs and access to services were highlighted as significant barriers to some especially for those living in countries where healthcare is not free at the point of delivery. Differing health care models mean services such as IVF are difficult to access in countries such as India and the USA, as they are usually not funded by the government and expensive to self-fund (20, 48). Besides pragmatic reasons, sociocultural attitudes towards PCOS in some communities deter those with PCOS from engaging in management plans. Carron et al. (20) discussed “fatalistic beliefs” associated with a diagnosis of PCOS in Native American communities. This cultivated a culture of self-blame and lack of hope, preventing women from seeking help from healthcare professionals (58, 59). Similarly, Hadjiconstantinou et al. (38) reported cultural taboos such as the use of oral contraceptives in “unmarried girls” inhibits uptake of PCOS management in migrant South Asian communities.

### 3.4. Perceptions

#### 3.4.1. Perception of the condition

Despite the high prevalence of psychological comorbidities associated with PCOS, many people reported adopting a positive outlook, with some choosing to take on exercise such as swimming, others through “positive reframing” of their mindset (18). Young et al. (35) found that many adolescents recognised the importance of being proactive in their own care and associated a positive outlook with decreased stress overall. A study of the effect of online peer support groups in women with PCOS found that many participants were more optimistic in their outlook after joining the support group (22). In a study by Tomlinson et al. (6), many women with PCOS found reading about others’ experience helped them overcome challenges. Furthermore, they felt that a positive outlook helped sustain personal motivations which is important in the management of a chronic condition.

#### 3.4.2. PCOS and society

PCOS as a condition itself challenges society’s expectations of femininity. Williams et al. (41) reported that symptoms associated with the condition, particularly irregular menstruation, hirsutism, and obesity made women with PCOS feel less feminine. On several occasions, these women referred to their symptoms with masculine terminology, such as describing their central adiposity as a “man’s beer belly”.

Those with symptoms of PCOS also found difficult to seek help from others in society. In a study by Sharma et al. (28), which took place in a traditional Indian society, the process of menstruation is considered taboo, with women reportedly being told by their mothers to avoid talking about menstrual issues and PCOS to others, particularly male relatives.

### 3.5. PCOS resources and improving outcomes

Ching et al. (53) reported from their study of 203 women with PCOS that increased satisfaction with quality of information received is associated with better health outcomes in women with PCOS. Studies reported the need for culturally specific education and treatment, not only for those with PCOS, but also their family and friends who may be unfamiliar with PCOS. It was stated for example, that educational outreach programs to produce tribal specific information would help to reduce marginalisation and stigmatisation of women with PCOS within tribes (20).

Avery et al. (7) found that culturally and locally relevant information improved engagement in some people with PCOS. One participant stated “That little flyer was really good. I liked it because it was Australian. I found out since then that lots of the stuff differs between America and Australia.”

Besides speaking to health care professionals, people with PCOS self-educate with resources including books, the internet, magazines and friends (54). However, these resources may not reliable. With an increase in information offered on the internet in particular, many people struggle to choose appropriate sources for PCOS-related information (7). Mallappa Saroja and Hanji Chandrashekhar (55) reviewed 15 PCOS related websites and concluded that none provided complete information on the condition.

## 4. Discussion

Our study details the symptoms which prompted people to seek care for PCOS and their experiences with diagnosis process. We also describe the various management strategies commonly employed in PCOS and the perceived barriers to access this management. This article also throws light on the perception of women with PCOS and society and how women cope with this. To the best of our knowledge, this is the first-of-its-kind systematic review detailing the various aspects of PCOS. The coding of all included articles by two independent researchers blinded by the objectives of the study bypassed the subjective bias. A further review of the codes by experienced qualitative researchers further strengthens the findings of the study.

While it is not a surprise to see the themes of menstrual irregularities, subfertility and skin related symptoms, we found emotional wellbeing with an intermix of body dysmorphia and weight stigma are increasingly being recognised by both women with PCOS and healthcare professionals. While a holistic approach in evaluating women with PCOS can help screen these concerns, its management may be limited by time limitations during consultations and availability of appropriate expertise.

There is a mixed experience with the diagnosis process. Research shows that education of healthcare professionals through increased training and engagement with current research improves PCOS management outcomes (56). This includes clinicians having greater experience within reproductive endocrinology and understanding both complex and standard presentations and management of PCOS.

While there are limited management options, our study reveals a spectrum of experiences and outcomes with both non-pharmacological and pharmacological therapy and reported interest in complementary and alternative therapies for PCOS. Overall, most people with PCOS were dissatisfied with the information and care provided by clinicians. Emphasis of this in clinical training is necessary as those with PCOS are at higher risk of mental health issues, and a better patient-doctor relationship will facilitate early diagnosis. It is necessary to Identify and address culturally specific concerns to ensure patients’ needs are holistically cared for. Follow-up care addressing the psychological wellbeing of those with PCOS is important, as those with PCOS are at greater risk of depressive symptoms.

There is a need to improve the perception of PCOS both at individual and societal levels. This can be achieved by evidence-based awareness programmes for all sections of the society. Conferences and seminars have been reported to be beneficial for patients and clinicians as they share current information on PCOS management (15). Currently, the main sources of information are clinicians, the internet, and books, although quality of information is inconsistent (39). Reviewing the contents and fact-checking the information can minimise misinformation and thus improve the perception and awareness about PCOS. Co-creation of resources with people with PCOS can ensure that the language used is easy to understand and addresses the knowledge gaps identified. Their impact should later be assessed by those with PCOS and their friends and families and adjusted accordingly. They should be updated regularly to ensure information is up to date.

Some of the strategies that may enhance holistic care for PCOS includes co-creation of healthcare services such as outpatient clinic design and accessibility. A pre-consultation survey with validated questionnaires regarding quality of life and emotional wellbeing will help in screening for the same. This can also get the people with PCOS start thinking about these areas and enquire about them during consultation. During consultation, patient preferences should be combined with clinical concerns which may increase patient satisfaction. A post-clinic survey about the experiences in the clinic may help identify areas of excellence and areas that need improving so the clinic can be finetuned to provide a better care. A regular survey of all staff involved in healthcare delivery can help understand their experiences and needs as well. Lastly, creation of educational resources should be based on current requirement of people of PCOS which can be identified by one-to-one meetings, focused group discussions and several online tools to identify hot topics of discussion. The drafted educational material should be reviewed by people with PCOS of various background and ethnicity to ensure it is culturally and socially acceptable. Future clinic services designed on these recommendations should be studied for their experiences to understand the benefits and limitations which can then help adopt similar practices worldwide.

While our systematic review provides an in-depth analysis to the current literature on patient perspective in PCOS, we acknowledge most studies included in our analysis collected data in high-income countries which predominantly speak English. In particular, we noted a lack of literature from primarily black or African nations. Therefore, this may not reflect the views from non-English speaking communities and globally elsewhere. Overall, there is paucity of data from low- and middle-income countries which we hope will be addressed by engaging and empowering researchers in those settings.

We also acknowledge we did not include non-scientists people with PCOS as part of our research teams. We felt this may introduce subjectivity and measurement bias if those people with PCOS introduce their personal views while coding the published literature. Instead, we included undergraduate students with none to minimal research background in the screening and coding of the systematic review. The students were also blinded to the objective of the study at the coding stage and thus established neutrality at the time of data collection and analysis.

## 5. Conclusion

There are mixed experiences and perceptions of those living with PCOS with the balance tipping more towards dissatisfaction. We identify a need to co-create resources which can enhance the knowledge of healthcare professional to provide culturally appropriate and holistic care for people with PCOS. This may be achieved by collaborative work between healthcare staff, professional societies, and patient support groups, both at the local and international level. Future studies should focus on exploring the various models of care available to identify and share best practices.

## Data availability statement

The original contributions presented in the study are included in the article/**Supplementary Material**. Further inquiries can be directed to the corresponding author.

## Author contributions

GL and ME conducted the searches and screened the articles to narrow down to full text inclusion. Both GL and ME were involved all stages of the study and have contributed equally to this work and share first authorship. MT and SI thematically analysed and coded the included full text articles. PL, CG, MO’R, and WA were involved in the design of the study and refining study methods. AL and PK conceptualised and supervised all stages of the study. They also critically analysed the codes to arrive at appropriate themes for results. Both AL and PK have contributed equally to this work and share senior authorship. Members of the PCOS SEva working group provided substantial contributions to the conception and design of the work study and were involved in discussions at all stages of the study. The group included Jameela Sheikh, Meghnaa Hebbar, Nawal Zia, Halimah Khalil, Saskia Wicks, Sindoora Jayaprakash, Alisha Narendran, Helena K. Gleeson, Lynne Robinson, Justin J. Chu, Tejal Lathia, Chitra Selvan, Carina Synn Cuen Pan, Kashish Malhotra, Eka Melson and Meri Davitadze. All authors contributed to the article and approved the submitted version.

## Funding

This work has been supported by the Wellcome Trust (Investigator Grant WT209492/Z/17/Z, to WA), the Health Research Board (Emerging Clinician Scientist Award ECSA-FA-2020-001, to MO’R) and The British Society for Paediatric and Adolescent Gynaecology (BritSPAG grant to PK). WA receives support from the NIHR Birmingham Biomedical Research Centre at the University Hospitals Birmingham NHS Foundation Trust and the University of Birmingham (Grant Reference Number BRC-1215-20009). The views expressed are those of the authors and not necessarily those of the NIHR UK or the Department of Health and Social Care UK.

## Acknowledgments

We thank Ms. Maureen Busby and members of DAISy-PCOS patient leaders group for inputs during the design of the study.

## Conflict of interest

The authors declare that the research was conducted in the absence of any commercial or financial relationships that could be construed as a potential conflict of interest.

## Publisher’s note

All claims expressed in this article are solely those of the authors and do not necessarily represent those of their affiliated organizations, or those of the publisher, the editors and the reviewers. Any product that may be evaluated in this article, or claim that may be made by its manufacturer, is not guaranteed or endorsed by the publisher.
