# Supplementary material for: A systematic review of lived experiences of people with polycystic ovary syndrome highlights the need for holistic care and co-creation of educational resources
Source: Front Endocrinol (Lausanne). 2022 Dec 2;13:1064937. doi: 10.3389/fendo.2022.1064937 (PMC9755159; doi:10.3389/fendo.2022.1064937)
Supplement: Supplementary file 1 [file DataSheet_1.docx]

Supplementary 1: Search strategy applied for the systematic review in Cochrane database

| ID | Search | Hits |
| --- | --- | --- |
| #1 | PCOS | 3292 |
| #2 | Polycystic ovary syndrome | 3687 |
| #3 | polycystic ovarian disease | 804 |
| #4 | Stein-Leventhal syndrome | 54 |
| #5 | {OR #1-#4} | 4331 |
| #6 | Education | 88667 |
| #7 | Health Education | 49524 |
| #8 | information | 1829732 |
| #9 | Health Information Exchange | 2143 |
| #10 | Consumer Health Information | 3529 |
| #11 | Access to Information | 33605 |
| #12 | Information Seeking Behavior | 3239 |
| #13 | Information Literacy | 4572 |
| #14 | Resources | 22006 |
| #15 | Health resources | 16990 |
| #16 | Video | 18899 |
| #17 | Video-Audio Media | 318 |
| #18 | {OR #6-#17} | 1829738 |
| #19 | Lived Experiences | 263 |
| #20 | opinion | 11328 |
| #21 | perspective | 12317 |
| #22 | viewpoint | 621 |
| #23 | comment | 14903 |
| #24 | attitude | 16297 |
| #25 | knowledge | 47741 |
| #26 | understanding | 24641 |
| #27 | comprehension | 2988 |
| #28 | Patient Medication Knowledge | 4115 |
| #29 | Health Knowledge | 28900 |
| #30 | Attitudes | 16487 |
| #31 | Practice | 109397 |
| #32 | {OR #19-#31} | 189954 |
| #33 | #5 and #18 and #32 | 91 |

Supplementary 2: Search strategy applied for the systematic review in Cochrane database

| 1 | (((PCOS) OR (Polycystic ovary syndrome)) OR (polycystic ovarian disease)) OR (Stein-Leventhal syndrome) | "PCOS"[All Fields] OR ("polycystic ovary syndrome"[MeSH Terms] OR ("polycystic"[All Fields] AND "ovary"[All Fields] AND "syndrome"[All Fields]) OR "polycystic ovary syndrome"[All Fields]) OR ("polycystic ovary syndrome"[MeSH Terms] OR ("polycystic"[All Fields] AND "ovary"[All Fields] AND "syndrome"[All Fields]) OR "polycystic ovary syndrome"[All Fields] OR ("polycystic"[All Fields] AND "ovarian"[All Fields] AND "disease"[All Fields]) OR "polycystic ovarian disease"[All Fields]) OR ("polycystic ovary syndrome"[MeSH Terms] OR ("polycystic"[All Fields] AND "ovary"[All Fields] AND "syndrome"[All Fields]) OR "polycystic ovary syndrome"[All Fields] OR ("stein"[All Fields] AND "leventhal"[All Fields] AND "syndrome"[All Fields]) OR "stein leventhal syndrome"[All Fields]) | 20,704 |
| --- | --- | --- | --- |
| 2 | (((((((((((Education) OR (Health Education)) OR (information)) OR (Health Information Exchange)) OR (Consumer Health Information)) OR (Access to Information)) OR (Information Seeking Behavior)) OR (Information Literacy)) OR (Resources)) OR (Health resources)) OR (Video)) OR (Video-Audio Media) | "educability"[All Fields] OR "educable"[All Fields] OR "educates"[All Fields] OR "education"[MeSH Subheading] OR "education"[All Fields] OR "educational status"[MeSH Terms] OR ("educational"[All Fields] AND "status"[All Fields]) OR "educational status"[All Fields] OR "education"[MeSH Terms] OR "education s"[All Fields] OR "educational"[All Fields] OR "educative"[All Fields] OR "educator"[All Fields] OR "educator s"[All Fields] OR "educators"[All Fields] OR "teaching"[MeSH Terms] OR "teaching"[All Fields] OR "educate"[All Fields] OR "educated"[All Fields] OR "educating"[All Fields] OR "educations"[All Fields] OR ("health education"[MeSH Terms] OR ("health"[All Fields] AND "education"[All Fields]) OR "health education"[All Fields]) OR ("inform"[All Fields] OR "informal"[All Fields] OR "informality"[All Fields] OR "informally"[All Fields] OR "informant"[All Fields] OR "informant s"[All Fields] OR "informants"[All Fields] OR "information"[All Fields] OR "information s"[All Fields] OR "informational"[All Fields] OR "informations"[All Fields] OR "informative"[All Fields] OR "informatively"[All Fields] OR "informativeness"[All Fields] OR "informativity"[All Fields] OR "informed"[All Fields] OR "informer"[All Fields] OR "informers"[All Fields] OR "informing"[All Fields] OR "informs"[All Fields]) OR ("health information exchange"[MeSH Terms] OR ("health"[All Fields] AND "information"[All Fields] AND "exchange"[All Fields]) OR "health information exchange"[All Fields]) OR ("consumer health information"[MeSH Terms] OR ("consumer"[All Fields] AND "health"[All Fields] AND "information"[All Fields]) OR "consumer health information"[All Fields]) OR ("access to information"[MeSH Terms] OR ("access"[All Fields] AND "information"[All Fields]) OR "access to information"[All Fields]) OR ("information seeking behavior"[MeSH Terms] OR ("information"[All Fields] AND "seeking"[All Fields] AND "behavior"[All Fields]) OR "information seeking behavior"[All Fields]) OR ("information literacy"[MeSH Terms] OR ("information"[All Fields] AND "literacy"[All Fields]) OR "information literacy"[All Fields]) OR ("health resources"[MeSH Terms] OR ("health"[All Fields] AND "resources"[All Fields]) OR "health resources"[All Fields] OR "resource"[All Fields] OR "resources"[All Fields] OR "resource s"[All Fields] OR "resourced"[All Fields] OR "resourceful"[All Fields] OR "resourcefulness"[All Fields] OR "resourcing"[All Fields]) OR ("health resources"[MeSH Terms] OR ("health"[All Fields] AND "resources"[All Fields]) OR "health resources"[All Fields]) OR ("video s"[All Fields] OR "videoed"[All Fields] OR "videotape recording"[MeSH Terms] OR ("videotape"[All Fields] AND "recording"[All Fields]) OR "videotape recording"[All Fields] OR "video"[All Fields] OR "videos"[All Fields]) OR ("video audio media"[Publication Type] OR "video audio media"[All Fields]) | 4,067,733 |
| 3 | (((((((((((Lived Experiences) OR (opinion)) OR (perspective)) OR (viewpoint)) OR (comment)) OR (attitude)) OR (knowledge)) OR (understanding)) OR (comprehension)) OR (Patient Medication Knowledge)) OR (Health Knowledge)) OR (Practice) | (("lived"[All Fields] OR "lives"[All Fields] OR "living"[All Fields] OR "livings"[All Fields]) AND ("experience"[All Fields] OR "experience s"[All Fields] OR "experiences"[All Fields])) OR ("attitude"[MeSH Terms] OR "attitude"[All Fields] OR "opinion"[All Fields] OR "opinions"[All Fields] OR "opinion s"[All Fields] OR "opinionated"[All Fields]) OR ("perspective"[All Fields] OR "perspective s"[All Fields] OR "perspectives"[All Fields]) OR ("comment"[Publication Type] OR "viewpoint"[All Fields]) OR ("comment"[Publication Type] OR "comment"[All Fields]) OR ("attitude"[MeSH Terms] OR "attitude"[All Fields] OR "attitudes"[All Fields] OR "attitude s"[All Fields]) OR ("knowledge"[MeSH Terms] OR "knowledge"[All Fields] OR "knowledge s"[All Fields] OR "knowledgeability"[All Fields] OR "knowledgeable"[All Fields] OR "knowledgeably"[All Fields] OR "knowledges"[All Fields]) OR ("comprehension"[MeSH Terms] OR "comprehension"[All Fields] OR "understand"[All Fields] OR "understanding"[All Fields] OR "understands"[All Fields] OR "understandability"[All Fields] OR "understandable"[All Fields] OR "understandably"[All Fields] OR "understandings"[All Fields]) OR ("comprehensibility"[All Fields] OR "comprehensible"[All Fields] OR "comprehension"[MeSH Terms] OR "comprehension"[All Fields] OR "comprehensions"[All Fields] OR "comprehensive"[All Fields] OR "comprehensively"[All Fields] OR "comprehensiveness"[All Fields]) OR ("patient medication knowledge"[MeSH Terms] OR ("patient"[All Fields] AND "medication"[All Fields] AND "knowledge"[All Fields]) OR "patient medication knowledge"[All Fields]) OR (("health"[MeSH Terms] OR "health"[All Fields] OR "health s"[All Fields] OR "healthful"[All Fields] OR "healthfulness"[All Fields] OR "healths"[All Fields]) AND ("knowledge"[MeSH Terms] OR "knowledge"[All Fields] OR "knowledge s"[All Fields] OR "knowledgeability"[All Fields] OR "knowledgeable"[All Fields] OR "knowledgeably"[All Fields] OR "knowledges"[All Fields])) OR ("practicability"[All Fields] OR "practicable"[All Fields] OR "practical"[All Fields] OR "practicalities"[All Fields] OR "practicality"[All Fields] OR "practically"[All Fields] OR "practicals"[All Fields] OR "practice"[All Fields] OR "practice s"[All Fields] OR "practiced"[All Fields] OR "practices"[All Fields] OR "practicing"[All Fields]) | 5,256,266 |
| 4 | (((((PCOS) OR (Polycystic ovary syndrome)) OR (polycystic ovarian disease)) OR (Stein-Leventhal syndrome)) AND ((((((((((((Education) OR (Health Education)) OR (information)) OR (Health Information Exchange)) OR (Consumer Health Information)) OR (Access to Information)) OR (Information Seeking Behavior)) OR (Information Literacy)) OR (Resources)) OR (Health resources)) OR (Video)) OR (Video-Audio Media))) AND ((((((((((((Lived Experiences) OR (opinion)) OR (perspective)) OR (viewpoint)) OR (comment)) OR (attitude)) OR (knowledge)) OR (understanding)) OR (comprehension)) OR (Patient Medication Knowledge)) OR (Health Knowledge)) OR (Practice)) | ("PCOS"[All Fields] OR ("polycystic ovary syndrome"[MeSH Terms] OR ("polycystic"[All Fields] AND "ovary"[All Fields] AND "syndrome"[All Fields]) OR "polycystic ovary syndrome"[All Fields]) OR ("polycystic ovary syndrome"[MeSH Terms] OR ("polycystic"[All Fields] AND "ovary"[All Fields] AND "syndrome"[All Fields]) OR "polycystic ovary syndrome"[All Fields] OR ("polycystic"[All Fields] AND "ovarian"[All Fields] AND "disease"[All Fields]) OR "polycystic ovarian disease"[All Fields]) OR ("polycystic ovary syndrome"[MeSH Terms] OR ("polycystic"[All Fields] AND "ovary"[All Fields] AND "syndrome"[All Fields]) OR "polycystic ovary syndrome"[All Fields] OR ("stein"[All Fields] AND "leventhal"[All Fields] AND "syndrome"[All Fields]) OR "stein leventhal syndrome"[All Fields])) AND ("educability"[All Fields] OR "educable"[All Fields] OR "educates"[All Fields] OR "education"[MeSH Subheading] OR "education"[All Fields] OR "educational status"[MeSH Terms] OR ("educational"[All Fields] AND "status"[All Fields]) OR "educational status"[All Fields] OR "education"[MeSH Terms] OR "education s"[All Fields] OR "educational"[All Fields] OR "educative"[All Fields] OR "educator"[All Fields] OR "educator s"[All Fields] OR "educators"[All Fields] OR "teaching"[MeSH Terms] OR "teaching"[All Fields] OR "educate"[All Fields] OR "educated"[All Fields] OR "educating"[All Fields] OR "educations"[All Fields] OR ("health education"[MeSH Terms] OR ("health"[All Fields] AND "education"[All Fields]) OR "health education"[All Fields]) OR ("inform"[All Fields] OR "informal"[All Fields] OR "informality"[All Fields] OR "informally"[All Fields] OR "informant"[All Fields] OR "informant s"[All Fields] OR "informants"[All Fields] OR "information"[All Fields] OR "information s"[All Fields] OR "informational"[All Fields] OR "informations"[All Fields] OR "informative"[All Fields] OR "informatively"[All Fields] OR "informativeness"[All Fields] OR "informativity"[All Fields] OR "informed"[All Fields] OR "informer"[All Fields] OR "informers"[All Fields] OR "informing"[All Fields] OR "informs"[All Fields]) OR ("health information exchange"[MeSH Terms] OR ("health"[All Fields] AND "information"[All Fields] AND "exchange"[All Fields]) OR "health information exchange"[All Fields]) OR ("consumer health information"[MeSH Terms] OR ("consumer"[All Fields] AND "health"[All Fields] AND "information"[All Fields]) OR "consumer health information"[All Fields]) OR ("access to information"[MeSH Terms] OR ("access"[All Fields] AND "information"[All Fields]) OR "access to information"[All Fields]) OR ("information seeking behavior"[MeSH Terms] OR ("information"[All Fields] AND "seeking"[All Fields] AND "behavior"[All Fields]) OR "information seeking behavior"[All Fields]) OR ("information literacy"[MeSH Terms] OR ("information"[All Fields] AND "literacy"[All Fields]) OR "information literacy"[All Fields]) OR ("health resources"[MeSH Terms] OR ("health"[All Fields] AND "resources"[All Fields]) OR "health resources"[All Fields] OR "resource"[All Fields] OR "resources"[All Fields] OR "resource s"[All Fields] OR "resourced"[All Fields] OR "resourceful"[All Fields] OR "resourcefulness"[All Fields] OR "resourcing"[All Fields]) OR ("health resources"[MeSH Terms] OR ("health"[All Fields] AND "resources"[All Fields]) OR "health resources"[All Fields]) OR ("video s"[All Fields] OR "videoed"[All Fields] OR "videotape recording"[MeSH Terms] OR ("videotape"[All Fields] AND "recording"[All Fields]) OR "videotape recording"[All Fields] OR "video"[All Fields] OR "videos"[All Fields]) OR ("video audio media"[Publication Type] OR "video audio media"[All Fields])) AND ((("lived"[All Fields] OR "lives"[All Fields] OR "living"[All Fields] OR "livings"[All Fields]) AND ("experience"[All Fields] OR "experience s"[All Fields] OR "experiences"[All Fields])) OR ("attitude"[MeSH Terms] OR "attitude"[All Fields] OR "opinion"[All Fields] OR "opinions"[All Fields] OR "opinion s"[All Fields] OR "opinionated"[All Fields]) OR ("perspective"[All Fields] OR "perspective s"[All Fields] OR "perspectives"[All Fields]) OR ("comment"[Publication Type] OR "viewpoint"[All Fields]) OR ("comment"[Publication Type] OR "comment"[All Fields]) OR ("attitude"[MeSH Terms] OR "attitude"[All Fields] OR "attitudes"[All Fields] OR "attitude s"[All Fields]) OR ("knowledge"[MeSH Terms] OR "knowledge"[All Fields] OR "knowledge s"[All Fields] OR "knowledgeability"[All Fields] OR "knowledgeable"[All Fields] OR "knowledgeably"[All Fields] OR "knowledges"[All Fields]) OR ("comprehension"[MeSH Terms] OR "comprehension"[All Fields] OR "understand"[All Fields] OR "understanding"[All Fields] OR "understands"[All Fields] OR "understandability"[All Fields] OR "understandable"[All Fields] OR "understandably"[All Fields] OR "understandings"[All Fields]) OR ("comprehensibility"[All Fields] OR "comprehensible"[All Fields] OR "comprehension"[MeSH Terms] OR "comprehension"[All Fields] OR "comprehensions"[All Fields] OR "comprehensive"[All Fields] OR "comprehensively"[All Fields] OR "comprehensiveness"[All Fields]) OR ("patient medication knowledge"[MeSH Terms] OR ("patient"[All Fields] AND "medication"[All Fields] AND "knowledge"[All Fields]) OR "patient medication knowledge"[All Fields]) OR (("health"[MeSH Terms] OR "health"[All Fields] OR "health s"[All Fields] OR "healthful"[All Fields] OR "healthfulness"[All Fields] OR "healths"[All Fields]) AND ("knowledge"[MeSH Terms] OR "knowledge"[All Fields] OR "knowledge s"[All Fields] OR "knowledgeability"[All Fields] OR "knowledgeable"[All Fields] OR "knowledgeably"[All Fields] OR "knowledges"[All Fields])) OR ("practicability"[All Fields] OR "practicable"[All Fields] OR "practical"[All Fields] OR "practicalities"[All Fields] OR "practicality"[All Fields] OR "practically"[All Fields] OR "practicals"[All Fields] OR "practice"[All Fields] OR "practice s"[All Fields] OR "practiced"[All Fields] OR "practices"[All Fields] OR "practicing"[All Fields])) | 541 |

Supplementary 3: Search strategy applied for the systematic review in Web of science database

| 1 | (((TS=(PCOS)) OR TS=(Polycystic ovary syndrome)) OR TS=(polycystic ovarian disease)) OR TS=(Stein-Leventhal syndrome) | [35,927](https://www.webofscience.com/wos/alldb/summary/ba1290d8-7fc5-4fed-944f-7d5396a06df9-06226171/relevance/1) |
| --- | --- | --- |
| 2 | (((((((((((TS=(Education)) OR TS=(Health Education)) OR TS=(Information)) OR TS=(Health information exchange)) OR TS=(consumer health information)) OR TS=(access to information)) OR TS=(information seeking behavior)) OR TS=(information literacy)) OR TS=(resources)) OR TS=(health resources)) OR TS=(video)) OR TS=(video-audio media) | [14,439,317](https://www.webofscience.com/wos/alldb/summary/20a3e205-5598-4aff-b43b-03399098fda8-062277a0/relevance/1) |
| 3 | ((((((((((((TS=(lived experiences)) OR TS=(opinion)) OR TS=(perspective)) OR TS=(viewpoint)) OR TS=(comment)) OR TS=(attitude)) OR TS=(knowledge)) OR TS=(understanding)) OR TS=(comprehension)) OR TS=(patient medication knowledge)) OR TS=(health knowledge)) OR TS=(attitudes)) OR TS=(practice) | [13,152,020](https://www.webofscience.com/wos/alldb/summary/dbf4d828-3222-4252-a2e9-74fdaa13adc3-06239320/relevance/1) |
| 4 | ((#1) AND #2) AND #3 | [481](https://www.webofscience.com/wos/alldb/summary/3b7282ef-5ed1-436b-a515-ca1806a44eab-06239c81/relevance/1) |

Supplementary 4: Search strategy applied for the systematic review in Psychinfo database

| 1 | PCOS.mp. | 322 |
| --- | --- | --- |
| 2 | Polycystic ovary syndrome.mp | 393 |
| 3 | polycystic ovarian disease.mp. | 8 |
| 4 | Stein-Leventhal syndrome.mp. | 2 |
| 5 | 1 or 2 or 3 or 4 | 450 |
| 6 | exp Education/ | 36223 |
| 7 | exp Health Education/ | 13811 |
| 8 | exp Information/ | 14548 |
| 9 | Health Information Exchange.mp. | 158 |
| 10 | Consumer Health Information.mp. | 999 |
| 11 | Access to Information.mp. | 3032 |
| 12 | Information Seeking Behavior.mp. | 1311 |
| 13 | exp Information Literacy/ | 333 |
| 14 | Resources.mp. | 133131 |
| 15 | Health resources.mp. | 2784 |
| 16 | Video.mp. | 46924 |
| 17 | Video-Audio Media.mp. | 2 |
| 18 | 6 or 7 or 8 or 9 or 10 or 11 or 12 or 13 or 14 or 15 or 16 or 17 | 242428 |
| 19 | Lived Experiences.mp. | 10292 |
| 20 | opinion.mp. | 33886 |
| 21 | perspective.mp. | 210286 |
| 22 | viewpoint.mp. | 11666 |
| 23 | comment.mp. | 27159 |
| 24 | attitude.mp. | 137441 |
| 25 | knowledge.mp. | 336311 |
| 26 | understanding.mp. | 406932 |
| 27 | exp Comprehension/ | 12909 |
| 28 | Patient Medication Knowledge.mp. | 33 |
| 29 | exp Health Knowledge/ | 8337 |
| 30 | exp Attitudes/ | 27561 |
| 31 | Practice.mp. | 402744 |
| 32 | 19 or 20 or 21 or 22 or 23 or 24 or 25 or 26 or 27 or 28 or 29 or 30 or 31 | 1265193 |
| 33 | 5 and 18 and 32 | 4 |

Supplementary 5: Search strategy applied for the systematic review in Medline database

| 1 | PCOS.mp. | 10406 |
| --- | --- | --- |
| 2 | Polycystic Ovary Syndrome/ | 15442 |
| 3 | polycystic ovarian disease.mp. | 634 |
| 4 | Stein-Leventhal syndrome.mp. | 737 |
| 5 | 1 or 2 or 3 or 4 | 16523 |
| 6 | Education/ or Education.mp. | 879527 |
| 7 | Health Education.mp. or Health Education/ | 86023 |
| 8 | information.mp. | 1170482 |
| 9 | Health Information Exchange.mp. or Health Information Exchange/ | 1563 |
| 10 | Consumer Health Information.mp. or Consumer Health Information/ | 4452 |
| 11 | Access to Information.mp. or Access to Information/ | 10096 |
| 12 | Information Seeking Behavior.mp. or Information Seeking Behavior/ | 3057 |
| 13 | Information Literacy.mp. or Information Literacy/ | 584 |
| 14 | resources.mp. | 250618 |
| 15 | Health Resources/ | 14338 |
| 16 | video.mp. | 130444 |
| 17 | Video-Audio Media/ | 31275 |
| 18 | 6 or 7 or 8 or 9 or 10 or 11 or 12 or 13 or 14 or 15 or 16 or 17 | 2238550 |
| 19 | lived experiences.mp. | 2500 |
| 20 | opinion.mp. | 85267 |
| 21 | perspective.mp. | 187326 |
| 22 | viewpoint.mp. | 25931 |
| 23 | Comment/ | 862579 |
| 24 | Attitude/ | 49350 |
| 25 | Knowledge/ or knowledge.mp. | 695995 |
| 26 | understanding.mp. | 760029 |
| 27 | Comprehension/ | 15713 |
| 28 | Patient Medication Knowledge/ | 202 |
| 29 | Knowledge/ or knowledge.mp. | 695995 |
| 30 | health knowledge.mp. | 120589 |
| 31 | attitudes.mp. | 201961 |
| 32 | practice.mp. | 990478 |
| 33 | 19 or 20 or 21 or 22 or 23 or 24 or 25 or 26 or 27 or 28 or 29 or 30 or 31 or 32 | 3262451 |
| 34 | 5 and 18 and 33 | 176 |

Supplementary 6: Search strategy applied for the systematic review in Embase database

| 1 | PCOS.mp. | 19326 |
| --- | --- | --- |
| 2 | Polycystic ovary syndrome.mp. | 19467 |
| 3 | polycystic ovarian disease.mp. | 848 |
| 4 | Stein-Leventhal syndrome.mp. | 385 |
| 5 | 1 or 2 or 3 or 4 | 25250 |
| 6 | Education.mp. or education/ | 1240297 |
| 7 | health education/ | 99558 |
| 8 | information.mp. or information/ | 2083423 |
| 9 | Health Information Exchange.mp. | 1225 |
| 10 | Consumer Health Information.mp. or consumer health information/ | 4529 |
| 11 | Access to Information.mp. or access to information/ | 25154 |
| 12 | Information Seeking Behavior.mp. | 730 |
| 13 | Information Literacy.mp. or information literacy/ | 902 |
| 14 | Resources.mp. | 315163 |
| 15 | Health resources.mp. | 7253 |
| 16 | Video.mp. | 161641 |
| 17 | Video-Audio Media.mp. | 13 |
| 18 | 6 or 7 or 8 or 9 or 10 or 11 or 12 or 13 or 14 or 15 or 16 or 17 | 3472871 |
| 19 | Lived Experiences.mp. | 3907 |
| 20 | opinion.mp. | 121996 |
| 21 | perspective.mp. | 291922 |
| 22 | viewpoint.mp. | 30517 |
| 23 | comment.mp. | 75079 |
| 24 | attitude/ | 67671 |
| 25 | knowledge/ | 44412 |
| 26 | understanding.mp. | 1140493 |
| 27 | comprehension/ | 32072 |
| 28 | Patient Medication Knowledge.mp. | 79 |
| 29 | Health Knowledge.mp. | 4947 |
| 30 | Attitudes.mp. | 155846 |
| 31 | Practice.mp. | 1727339 |
| 32 | 19 or 20 or 21 or 22 or 23 or 24 or 25 or 26 or 27 or 28 or 29 or 30 or 31 | 3356146 |
| 33 | 5 and 18 and 32 | 322 |
